# Assessing the stability of psychobiological stress reactivity during adolescence: mixed-effect modelling of cortisol responses to laboratory stressors

**DOI:** 10.12688/hrbopenres.13874.2

**Published:** 2025-01-24

**Authors:** Jen O'Shea, Samantha Dockray, Elizabeth Susman

**Affiliations:** 1 School of Applied Psychology, University College Cork, Cork, County Cork, T12 K8AF, Ireland; 2 Department of Biobehavioral Health, The Pennsylvania State University - University Park Campus, University Park, Pennsylvania, PA 16802, USA

**Keywords:** Stress, stress reactivity, puberty, pubertal development, pubertal timing, pubertal tempo, longitudinal

## Abstract

**Background:**

Puberty has been historically considered as a time of risk and vulnerability for young people. It is associated with rapid development in the hypothalamus, which is central in the production of both stress and sex steroids. While patterns of stress reactivity are calibrated in early life, this time of rapid development may provide a means for these patterns to change. This purpose of this study was to examine whether patterns of cortisol reactivity remained stable across one year of pubertal development, and whether variations in pubertal development impacted on this stability.

**Methods:**

This study used a secondary dataset comprised of 102 adolescent-aged children and adolescents. Children and adolescents took part in the Trier Social Stress Test to elicit a physiological stress response. Cortisol reactivity was measured as the increase in salivary cortisol concentration taken at five time points throughout the session. Pubertal stage was measured by nurse report where possible, and parent/self-report otherwise and was used to calculate pubertal timing and tempo relative to peers. Measures of anxiety, BMI, and socio-economic status were taken and included in analysis.

**Results:**

Results of a linear mixed-effect model found there to be a significant difference in cortisol reactivity over time, indicating that cortisol stress reactivity did not remain stable during this time (Estimate= 3.39, t=3.67, p<.001, CI[1.56, 5.22]). Additionally, results show children and adolescents who developed slower/quicker than peers displayed decreased stress reactivity (Estimate= -3.59, t=-2.13. p=.03, CI[-6.92, -0.25]).

**Conclusions:**

This research contributes to a relatively small but consistent body of research noting pattern of increased cortisol reactivity during pubertal development. While a significant effect was found for pubertal tempo, this finding should not be considered indicative of any true effect.

## Introduction

Experiences and events that represent a challenge to a person typically elicit coping responses characterised by a pattern of neuroendocrine and behavioural changes. These changes include the stress response system, and the activation of the hypothalamic-pituitary-adrenal axis, with subsequent changes in cortisol. This change in cortisol, described as cortisol reactivity, has been associated with several dimensions of physical and psychological wellbeing across the lifespan ( Turner *et al.*, 2020). People with excessive, blunted and/or persistently high cortisol responses to stress, or individuals who are exposed to chronic and persistent stress responses are at risk of negative physical, emotional, and behavioural health issues ( Adam *et al.*, 2017; Beckie, 2012; Dickerson & Kemeny, 2004) as cortisol has effects across all body systems. Patterns of cortisol reactivity are calibrated by experiences and environmental context in early life, as information about environmental threat and opportunities are embedded in setpoints and reactivity patterns of psychobiological systems ( Ellis *et al.*, 2017). Recent evidence indicates that re-calibration of the cortisol stress response is possible ( DePasquale *et al.*, 2019; Engel & Gunnar, 2020; Gunnar *et al.*, 2019; Peckins *et al.*, 2015) and this plasticity may be enabled via the maturational processes occurring in the neural and endocrine systems at adolescence ( DePasquale *et al.*, 2021). The period of maturation during adolescence can open windows of opportunity that may reconfigure vulnerabilities linked to a recalibration of cortisol stress reactivity, and puberty has been highlighted as a biopsychosocial process that can reconfigure stress reactivity ( Worthman *et al.*, 2019).

### Pubertal development

Puberty is a physiological process that enables and prompts change in the psychological and social life of the person; these biopsychosocial changes have been considered within both risk and protective frameworks ( Chen & Raine, 2018; Hummel *et al.*, 2013). The focus has often been on pubertal timing, or measurement of when children and adolescents reach specific physiological, hormonal, or other domain-dependent stages of pubertal development relative to their same age and sex peers ( Mendle *et al.*, 2010). Earlier or later onset of puberty has been linked to future and concurrent mental and physical health, for example, people, particularly girls, who commence puberty ahead of their peers are at greater risk of experiencing affect disorders ( Galvao *et al.*, 2014), behavioural problems ( Dimler & Natsuaki, 2015), and health issues in later life ( Day *et al.*, 2015). Similar associations between earlier pubertal onset and adverse outcomes have also been observed in boys ( Ullsperger & Nikolas, 2017), however, these negative outcomes may also be largely shaped by social contextual factors. For example, for boys with less harsh, more supportive parenting, earlier onset of puberty has been associated with enhanced social competence and positive psychosocial development, as well less aggressive behaviour ( Klopack *et al.*, 2019). In girls, timing has been shown to only predict aggressive behaviour when coupled with low maternal nurturance ( Mrug *et al.*, 2008). The interaction of parental relationships and pubertal timing has been observed in both boys and girls, with positive parenting moderating the relationship between off-time pubertal onset and adverse outcomes ( Chen & Raine, 2018). These findings support the concept that health and behavioural outcomes are not solely determined by the physiological processes, or the timing or tempo of these alone, but rather situated within the context of the young person’s environment.

The impact of pubertal development on adolescent development has been examined in combination with stress responsivity, including as indexed by cortisol reactivity, and symptoms of depression have been predicted by an interaction of earlier puberty and heightened stress reactivity ( Gong *et al.*, 2019). Interestingly, one study found there to be a link between the timing of puberty, the patterns of cortisol reactivity, and the risk of depressive symptoms ( Colich *et al.*, 2015). Here, depressive symptoms were predicted by HPA hyporeactivity in those who began puberty earlier than their peers, and HPA hyperreactivity in those who had begun puberty later. What these studies may indicate, is that the timing of puberty is a complex, highly socially contextual process that is closely linked with outcomes related to stress.

Whereas pubertal timing is the onset of puberty relative to peers, pubertal tempo refers to the rate at which an individual progresses through puberty. There has been comparatively little research exploring the influence of pubertal tempo on biopsychosocial development, however research to date suggests that relatively fast or slow pubertal tempo may be associated with both depressive symptoms ( Keenan *et al.*, 2014; Mendle, 2014) and behavioural issues ( Marceau *et al.*, 2011). One recent systematic review of outcomes related to pubertal tempo found that while both boys and girls who experienced accelerated development had symptoms of depression during childhood and adolescence, there were distinct gender differences ( Cheng *et al.*, 2020). For instance, boys tended to experience more psychosocial difficulties due to accelerated development, for girls this was an opportunity to compensate for the impact of later onset puberty. These findings can be somewhat contradictory, and indicate there may be strong gender differences in the risk and protective factors associated with development ( Mendle *et al.*, 2010). In the context of cortisol reactivity, there is relatively little research examining the impact of pubertal tempo, however, accelerated development is associated with heightened stress reactivity, and this reactivity is associated with later depressive symptoms ( Gong *et al.*, 2019). It is important to note that while pubertal development and HPA maturation is associated with negative outcomes, some suggest that these should be treated as neither antecedents nor consequences of development, but rather as contributors to a system of accumulating risk, such as with allostatic load ( Joos *et al.*, 2018; Whelan *et al.*, 2021). In other words, where pubertal timing and pubertal tempo can influence cortisol reactivity patterns, these exist within a wide system of opportunities for positive and negative growth.

### Stability of cortisol reactivity during pubertal development

Patterns of cortisol secretion are reported to change during adolescence, with a normative increase in cortisol production, and differences in diurnal cortisol and cortisol reactivity partly explained by pubertal development, and differences in age and sex groups noted ( Gunnar *et al.*, 2009; Ji *et al.*, 2016; Platje *et al.*, 2013; Rotenberg *et al.*, 2012). Because of this, adolescence provides a unique opportunity to study stability, as stability/instability is highly linked to this rapid development in the Hypothalamic Adrenal Pituitary axis, and the Hypothalamic Pituitary Gonadal axis, and offers a unique opportunity to study stability and change ( Gunnar *et al*., 2019). The Pubertal Stress Recalibration Hypothesis (PSHR) describes the process wherein several bioecological and neurological factors, such as heightened plasticity in the hypothalamus, association cortex, and prefrontal cortex, and heightened sensitivity to socio-emotional environments, may allow for physiological responses to stress to be recalibrated during adolescence. There is some evidence from both cross-sectional ( DePasquale *et al.*, 2019; Zhang *et al.*, 2021) and longitudinal ( Gunnar *et al.*, 2019) studies to indicate a recalibration of cortisol responses in the context of positive social environments, at least in children and adolescents who experienced institutionalisation. However, there has been limited exploration of how cortisol reactivity or stability may be impacted by off-timing or off-tempo pubertal development. It is suggested that cortisol reactivity is influenced by the timing of puberty, with greater associations between off-time development and moderations in both stress reactivity and recovery ( Smith & Powers, 2009). The current study examines the potential role of off-time and off-tempo pubertal development on the stability of cortisol reactivity to laboratory stress challenge across a period of adolescent development.

## Methods

### Participants

This study uses secondary data comprised of a sample of 135 participants collected as part of the 'Physiology of Puberty and Antisocial Behavior' project (NIMH:5R01MH058393-03). Initially, a comprehensive list of all children residing in specific zip code areas was obtained from the American Student List (ASL), a commercial organization that provides names of school-aged children and adolescents. The zip codes chosen belonged to the county where the research was conducted, as well as adjacent counties that were easily accessible to the laboratory. This recruitment process was inclusive of rural and semi-rural addresses located within a reasonable distance to the laboratory. A letter was sent to 966 parents, requesting their cooperation in the study and to be contacted by the research team. Following this, the research staff personally reached out to the parents through phone calls, seeking the child / adolescents' participation. Ultimately, 85 children and adolescents were enrolled based on the responses from their parents. Among the remaining children and adolescents, 584 could not be contacted due to returned letters and lack of forwarding contact information, while 89 others did not meet the inclusion criteria. Additional participants were recruited via flyers distributed throughout the community and through telephone responses to emails sent to university staff members. All these recruitment procedures were approved by the Institutional Review Board at Pennsylvania State University.

This data set is comprised of biological, physiological, and psychological measures. Of the 135 included in the final data set, thirty-three were removed due to missing data in cortisol reactivity or pubertal measured. Participants included in the current study include 102 (49 boys, 53 girls) adolescent-aged children and adolescents. At the first wave of measurement, girls were aged 8, 10 or 12 years (Mean = 10.96, SD=1.66), and boys were aged 9, 11 or 13 years (Mean = 10.08, SD = 1.66). The difference in ages between sexes was to account for noted differences in the typical time young people enter puberty. All participants included in this study were free from chronic health problems and were not using any medication known to interfere with hormone levels. Participants were predominantly white (non-Hispanic) (94%), Hispanic (4%), African American (4%), and Asian (1).

A more detailed breakdown of this sample has been described previously ( Susman *et al*., 2010), however, a summary of these details can be seen in the table below:

**Table 1.** Descriptive Statistics.

	Wave 1	Wave 2	Wave 3
Mean Age (SD)	10.51 (1.7)	10.94 (1.7)	11.64 (1.7)
Age Range (lower, upper)	8, 13	8, 14	9, 15
Mean days elapsed since previous wave (SD)	-	197.6 (24.1)	186.4 (29.9)

This data set is comprised of biological, physiological and psychological measures collected over the course of three years. Participants included in the current study include 102 (49 boys, 53 girls) adolescent-aged children. At the first wave of measurement, girls were aged 8, 10 or 12 years (Mean = 10.96, SD=1.66), and boys were aged 9, 11 or 13 years (Mean = 10.08, SD = 1.66). All participants included in this study were free from chronic health problems and were not using any medication known to interfere with hormone levels.

### Dependent variable

***Cortisol reactivity.*** Cortisol reactivity was measured using salivary cortisol levels in response to the Trier Social Stress Test for Children/Adolescents (TSST-C) ( Allen *et al.*, 2017). The TSST-C is a test which elicits a stress response in participants by using both social evaluative and cognitive components. Children and adolescents were asked to come up with the end to a story in front of two judges and told that their answer would be compared to those of other children and adolescents their age. Prior to the commencement of the test, two samples were gathered to estimate a baseline measure of cortisol. After the administering of the task, three further samples were collected, first immediately after the task, then in ten-minute intervals. Cortisol reactivity is operationalised as the cortisol response to stress, measured as Area Under the Curve with respect to increase (AUC _*I*_) ( Pruessner *et al.*, 2003). This method provides a measure of change over time, as it ignores the distance from zero on all measurements, instead using baseline measures as a focal point for increase, and is thus more useful for increase response and acute measurement ( Fekedulegn *et al.*, 2007). A breakdown of demographics can be seen in Table 1. Previous research has opted to use a larger scale growth curve analysis to calculate cortisol response and recovery ( Ji *et al.*, 2016). These calculations provide a sensitive and robust model. However, this research is concerned with the overall production of cortisol response, relative to the baseline and so AUCi ( Pruessner *et al*., 2003) was used. 

### Independent variables

***Pubertal timing and tempo.*** Pubertal timing and tempo were calculated using pubertal stage. Pubertal stage was assessed by a paediatric research nurse during the research visit, and was operationalised following Tanner guidelines of genital stage and pubic hair stage for boys, and breast stage and pubic hair for girls ( Marshall & Tanner, 1968), and included a self-report, parent report, and physical exam. To achieve this, the nurse first explained to the participants what each of the five stages of puberty included. The nurse then showed the parent and adolescent pictures of each of the five Tanner stages, and asked them to independently identify what stage of development the adolescent had reached. For this study, participant self-report of stage (genital for boys, breast for girls) was used in the analysis. Pubertal timing was calculated using a regression of pubertal stage on age of participants stratified by gender ( Dorn *et al.*, 2003; Dorn *et al.*, 2006). The resulting residual value was then used as an index of pubertal timing – where a higher value indicates a later timing of puberty, and a lower value indicates an earlier timing of puberty. Pubertal tempo was calculated using linear growth curve estimation. This method was based on an adapted version of previous methods used ( Marceau *et al.*, 2011). To calculate individual tempo, each growth trajectory was modelled using linear growth curve estimation, and the total variance over time was used as an indicator of pubertal tempo.

***Anxiety.*** Anxiety was assessed using participants Anxious/Depressed scores on the subcategory of the Child Behaviour Checklist (CBCL) ( Achenbach & Edelbrock, 1991). The CBCL is a checklist which measures problem behaviour and emotional problems in children and adolescents, and has been well-validated cross-culturally ( Ivanova *et al.*, 2007). The CBCL was completed by parents / guardians of the child and the parent data was used in this analysis.

***Covariates.*** There are several other factors that may be relevant in examining the association of pubertal development with cortisol reactivity profiles, including Socio-economic status (SES) ( Reiss, 2013; Vliegenthart *et al.*, 2016), body-mass index (BMI) ( Dockray *et al.*, 2009), and sex. SES was determined using the Hollingshead four-factor index of social status ( Hollingshead, 1975). The mean score for participants was 47 (SD = 12), out of a possible 66. BMI was calculated using the standard formula of weight (km)/height² (m²) at each wave of measurement. Measurements of height and weight were obtained through nurse reports during a physical exam, with an average of three measurements taken as the operant value.

***Statistical analysis.*** All data analyses were conducted using the IBM Statistical Package for the Social Sciences (SPSS), version 28. A linear mixed-effect model was used to examine whether there were any associations between cortisol reactivity to stress and each of the independent variables between each wave of measurement ( West, 2009). The effects of SES, BMI, and sex were controlled for in the model. A summary of mean values of age, pubertal timing, pubertal tempo, cortisol reactivity, BMI, and anxiety can be seen in Table 2 below.

**Table 2.** Results and descriptives.

	Mean			SD		
	*Overall*	*Boys*	*Girls*	*Overall*	*Boys*	*Girls*
**Age**	10.5	10.96	10.08	1.71	1.65	1.65
**Pubertal timing**	-.151	-.17	-.134	.737	.644	.815
**Pubertal tempo**	.564	.398	.717	.764	.536	.901
	*Wave 1*	*Wave 2*	*Wave 3*	*Wave 1*	*Wave 2*	*Wave 3*
**Cortisol Reactivity**	-2.02	2.71	4.23	3.89	6.95	7.12
**BMI**	20.05	20.64	21.28	4.7	4.96	6.34
**CBCL Anxiety**	2.22	1.67	1.56	2.95	2.16	2.19

## Results

### Stability of cortisol reactivity across adolescence

There was a significant difference in cortisol reactivity to stress between timepoints (Estimate= 3.39, t=3.67, p<.001, CI[1.56, 5.22]). We observed a general trend of increasing cortisol reactivity from wave 1 (Mean=-2.02, SD=3.89) to wave 3 (Mean = 4.23, SD =7.12) of data collection (Estimate=3.39, t=3.67, CI[1.56, 5.22]) (see Table 3).

**Table 3.** Results of the mixed-effect linear model.

**Parameter**	**Estimate**	**Std Error**	**df**	**t**	**P**	**Lower CI**	**Upper CI**
**Time**	3.38891	.924304	115.918	3.666	.000*	1.55819	5.21962
**Pubertal Timing**	2.70507	1.70016	133.679	1.591	.114	-.657630	6.06777
**Pubertal Tempo**	-3.58567	1.68430	123.009	-2.129	.035*	-6.91964	-.251696
**CBCL Anxiety**	-.023065	.341428	150.678	-.068	.946	-.69767	.651539

### Impact of pubertal timing and tempo on this relationship

There was no direct significant effect of pubertal timing or anxiety on cortisol reactivity profiles over time, although we did observe a significant main effect of pubertal tempo on cortisol activity (Estimate= -3.59, t=-2.13. p=.03, CI[-6.92, -0.25]). We found that more rapid pubertal tempo was associated with a reduced cortisol reactivity profile, however the width of confidence interval limits meaningful inference.

## Discussion

Historically, it was generally accepted that cortisol production remains relatively stable during puberty, displaying a normative pattern of increase throughout development with factors such as age and sex influencing differentiations in cortisol stability ( Gunnar *et al.*, 2009; Ji *et al.*, 2016; Platje *et al.*, 2013; Rotenberg *et al.*, 2012). This time was conceptualised as a period of risk and vulnerability, where children and adolescents were susceptible to negative orientations in physiological responses to stress risk and the development of psychopathologies ( Roberts & Lopez-Duran, 2019). There is an emerging perspective within developmental psychobiology which states that while there is increased risk and vulnerability during the adolescent transition, this time may also be conceived as a time of opportunity and positive development. The purpose of this study was to investigate whether cortisol response to stressors, as a measure of stress reactivity, remained stable across a period of pubertal development. Additionally, this study aimed to examine whether pubertal timing and/or pubertal tempo influenced any observed change in reactivity while controlling for anxiety. We found that cortisol reactivity was not stable across the timepoints of measurement, observing a general trend wherein cortisol reactivity increased over time. This result is consistent with the normative pattern of change in cortisol secretion during adolescence development ( Gunnar *et al.*, 2009; Platje *et al.*, 2013; Rotenberg *et al.*, 2012). Importantly, previous research examining the stability of cortisol reactivity during adolescence has found similar results, despite using a separate analytic method. This is likely due to this normative pattern of increasing initial response, and variations in response.

There was no significant direct impact of either pubertal timing, or pubertal tempo on cortisol reactivity indicated in the results. One possible reason for this is the homogeneity of the sample used; children and adolescents in this study shared similar backgrounds, few could be considered as disadvantaged. This is important as contemporary literature suggests that the current social environments of young people plays a crucial role in determining later developmental outcomes ( DePasquale *et al.*, 2019). Here, regardless of whether there has been experience of adversity in early life, the coupling of rapid development during puberty with positive social environments provides an opportunity for recalibration of previously atypical responses to stressors ( Gunnar *et al.*, 2019; Zhang *et al.*, 2021). For these reasons, these results should not be considered indicative of the lack of relationship between pubertal development and cortisol reactivity stability, and future research is needed using a more diverse sample to further examine this relationship.

We acknowledge that the study has limitations which require caution in considering the findings. Firstly, the sample used in this study is both too small, and too homogenous to derive any indication of the true effect in the population. This is important when considering the differences in both stress reactivity and puberty that may be associated with social contextual or individual aspects such as SES. Secondly, the total time difference between the first and final wave collection was one year. While this is theoretically enough to observe some pattern of instability, there is a potential loss of variability, and this method cannot account for variability of pubertal tempo. It is possible that in this sample that variability in change existed across the time points, and thus, more research is warranted investigating the stability of acceleration (and deceleration) of puberty, and how this may impact upon cortisol reactivity. One strategy with potential to do this is to integrate different measures of pubertal timing ( Dorn *et al.*, 2006; Mendle, 2014), which would allow for. One of the primary strengths of this study, however, is that to the best of our knowledge, no longitudinal research has been conducted which investigates the direct relationship of both pubertal timing and tempo on the stability of cortisol reactivity and this study provides a foundation for further research.

In summary, we found that cortisol reactivity was not stable across time points. While our secondary hypothesis, that pubertal timing and tempo would significantly influence this instability was not supported, the results suggest that off-time or off-tempo pubertal development should not necessarily be considered as singular, or interactive, predictors of atypical cortisol stress reactivity to stress. Instead, it is more likely that these factors exert effects within a complex biopsychosocial system of risk and protection during adolescence. These findings are therefore important to the context of developing practical intervention programmes for young people, as literature suggests there is a strong association between the timing of these positive social environments and later outcomes For researchers studying the effect of experience, including young people’s engagement with enrichment and other programmes, it is important to consider how psychobiological measures of stress may change as much via pubertal development, as they may as a response to the programme or intervention. Looking forward, further focus should be placed on identifying and understanding the interconnected biological, physiological and environmental mechanisms which underpin this normative process of cortisol reactivity instability and change.

## Data Availability

Data are not available for this study. This study used a secondary data set where consent was not provided in the original study to allow sharing or access to third parties. Data were obtained from the 'Physiology of Puberty and Antisocial Behavior' project (NIMH:5R01MH058393-03). Any further requests should be made to the original research team.

## References

[ref-1] Achenbach TM Edelbrock C : Child behavior checklist. *Burlington (Vt).* 1991;7:371–392.

[ref-2] Adam EK Quinn ME Tavernier R : Diurnal cortisol slopes and mental and physical health outcomes: a systematic review and meta-analysis. *Psychoneuroendocrinology.* 2017;83:25–41. 10.1016/j.psyneuen.2017.05.018 28578301 PMC5568897

[ref-3] Allen AP Kennedy PJ Dockray S : The trier social stress test: principles and practice. *Neurobiol Stress.* 2017;6:113–126. 10.1016/j.ynstr.2016.11.001 28229114 PMC5314443

[ref-4] Beckie TM : A systematic review of Allostatic Load, health, and health disparities. *Biol Res Nurs.* 2012;14(4):311–346. 10.1177/1099800412455688 23007870

[ref-5] Chen FR Raine A : Effects of harsh parenting and positive parenting practices on youth aggressive behavior: the moderating role of early pubertal timing. *Aggress Behav.* 2018;44(1):18–28. 10.1002/ab.21720 28699265

[ref-6] Cheng HL Harris SR Sritharan M : The tempo of puberty and its relationship to adolescent health and well-being: a systematic review. *Acta Paediatr.* 2020;109(5):900–913. 10.1111/apa.15092 31730292

[ref-7] Colich NL Kircanski K Foland-Ross LC : HPA-axis reactivity interacts with stage of pubertal development to predict the onset of depression. *Psychoneuroendocrinology.* 2015;55:94–101. 10.1016/j.psyneuen.2015.02.004 25745954 PMC4380614

[ref-8] Day FR Elks CE Murray A : Puberty timing associated with diabetes, cardiovascular disease and also diverse health outcomes in men and women: the UK Biobank study. *Sci Rep.* 2015;5: 11208. 10.1038/srep11208 26084728 PMC4471670

[ref-9] DePasquale CE Donzella B Gunnar MR : Pubertal recalibration of cortisol reactivity following early life stress: A cross-sectional analysis. *J Child Psychol Psychiatry.* 2019;60(5):566–575. 10.1111/jcpp.12992 30357830 PMC6458083

[ref-10] DePasquale CE Herzberg MP Gunnar MR : The pubertal stress recalibration hypothesis: potential neural and behavioral consequences. *Child Dev Perspect.* 2021;15(4):249–256. 10.1111/cdep.12429 34925549 PMC8680280

[ref-11] Dickerson SS Kemeny ME : Acute stressors and cortisol responses: a theoretical integration and synthesis of laboratory research. *Psychol Bull.* 2004;130(3):355–391. 10.1037/0033-2909.130.3.355 15122924

[ref-12] Dimler LM Natsuaki MN : The effects of pubertal timing on externalizing behaviors in adolescence and early adulthood: a meta-analytic review. *J Adolesc.* 2015;45(1):160–170. 10.1016/j.adolescence.2015.07.021 26439868

[ref-13] Dockray S Susman EJ Dorn LD : Depression, cortisol reactivity, and obesity in childhood and adolescence. *J Adolesc Health.* 2009;45(4):344–350. 10.1016/j.jadohealth.2009.06.014 19766938 PMC2750854

[ref-14] Dorn LD Dahl RE Woodward HR : Defining the boundaries of early adolescence: a user’s guide to assessing pubertal status and pubertal timing in research with adolescents. *Appl Dev Sci.* 2006;10(1):30–56. 10.1207/s1532480xads1001_3

[ref-15] Dorn LD Susman EJ Ponirakis A : Pubertal timing and adolescent adjustment and behavior: conclusions vary by rater. *J Youth Adolesc.* 2003;32:157–167. 10.1023/A:1022590818839

[ref-16] Ellis BJ Oldehinkel AJ Nederhof E : The Adaptive Calibration Model of stress responsivity: an empirical test in the Tracking Adolescents’ Individual Lives Survey study. *Dev Psychopathol.* 2017;29(3):1001–1021. 10.1017/S0954579416000985 27772536

[ref-17] Engel ML Gunnar MR : The development of stress reactivity and regulation during human development. *Int Rev Neurobiol.* Elsevier,2020;150:41–76. 10.1016/bs.irn.2019.11.003 32204834

[ref-18] Fekedulegn DB Andrew ME Burchfiel CM : Area under the curve and other summary indicators of repeated waking cortisol measurements. *Psychosom Med.* 2007;69(7):651–659. 10.1097/PSY.0b013e31814c405c 17766693

[ref-19] Galvao TF Silva MT Zimmermann IR : Pubertal timing in girls and depression: a systematic review. *J Affect Disord.* 2014;155:13–19. 10.1016/j.jad.2013.10.034 24274962

[ref-20] Gong C Duan X Su P : Heightened HPA-axis stress reactivity and accelerated pubertal progression predicts depressive symptoms over 4-year follow up. *Psychoneuroendocrinology.* 2019;103:259–265. 10.1016/j.psyneuen.2019.02.001 30754000

[ref-21] Gunnar MR DePasquale CE Reid BM : Pubertal stress recalibration reverses the effects of early life stress in postinstitutionalized children. *Proc Natl Acad Sci U S A.* 2019;116(48):23984–23988. 10.1073/pnas.1909699116 31712449 PMC6883847

[ref-22] Gunnar MR Wewerka S Frenn K : Developmental changes in hypothalamus-pituitary-adrenal activity over the transition to adolescence: normative changes and associations with puberty. *Dev Psychopathol.* 2009;21(1):69–85. 10.1017/S0954579409000054 19144223 PMC3933029

[ref-23] Hollingshead AB : Four factor index of social status. 1975. Reference Source

[ref-24] Hummel A Shelton KH Heron J : A systematic review of the relationships between family functioning, pubertal timing and adolescent substance use. *Addiction.* 2013;108(3):487–496. 10.1111/add.12055 23163243

[ref-25] Ivanova MY Dobrean A Dopfner M : Testing the 8-syndrome structure of the child behavior checklist in 30 societies. *J Clin Child Adolesc Psychol.* 2007;36(3):405–417. 10.1080/15374410701444363 17658984

[ref-26] Ji J Negriff S Kim H : A study of cortisol reactivity and recovery among young adolescents: heterogeneity and longitudinal stability and change. *Dev Psychobiol.* 2016;58(3):283–302. 10.1002/dev.21369 26517401

[ref-27] Joos CM Wodzinski AM Wadsworth ME : Neither antecedent nor consequence: developmental integration of chronic stress, pubertal timing, and conditionally adapted stress response. *Developmental Review.* 2018;48:1–23. 10.1016/j.dr.2018.05.001

[ref-28] Keenan K Culbert KM Grimm KJ : Timing and tempo: exploring the complex association between pubertal development and depression in African American and European American girls. *J Abnorm Psychol.* 2014;123(4):725–736. 10.1037/a0038003 25314262 PMC4227930

[ref-29] Klopack ET Sutton TE Simons RL : Disentangling the effects of boys’ pubertal timing: the importance of social context. *J Youth Adolesc.* 2019;49(7):1393–1405. 10.1007/s10964-019-01141-9 31587175 PMC9290439

[ref-30] Marceau K Ram N Houts RM : Individual differences in boys’ and girls’ timing and tempo of puberty: modeling development with nonlinear growth models. *Dev Psychol.* 2011;47(5):1389–1409. 10.1037/a0023838 21639623 PMC3928626

[ref-31] Marshall WA Tanner JM : Growth and physiological development during adolescence. *Annu Rev Med.* 1968;19(1):283–300. 10.1146/annurev.me.19.020168.001435 4297619

[ref-32] Mendle J : Beyond pubertal timing: new directions for studying individual differences in development. *Curr Dir Psychol Sci.* 2014;23(3):215–219. 10.1177/0963721414530144

[ref-33] Mendle J Harden KP Brooks-Gunn J : Development’s tortoise and hare: pubertal timing, pubertal tempo, and depressive symptoms in boys and girls. *Dev Psychol.* 2010;46(5):1341–53. 10.1037/a0020205 20822243 PMC3114603

[ref-34] Mrug S Elliott M Gilliland MJ : Positive parenting and early puberty in girls: protective effects against aggressive behavior. *Arch Pediatr Adolesc Med.* 2008;162(8):781–786. 10.1001/archpedi.162.8.781 18678812

[ref-35] Peckins MK Susman EJ Negriff S : Cortisol profiles: a test for adaptive calibration of the stress response system in maltreated and nonmaltreated youth. *Dev Psychopathol.* 2015;27(4 pt 2):1461–1470. 10.1017/S0954579415000875 26535937 PMC5353977

[ref-36] Platje E Vermeiren RR Branje SJ : Long-term stability of the Cortisol Awakening Response over adolescence. *Psychoneuroendocrinology.* 2013;38(2):271–280. 10.1016/j.psyneuen.2012.06.007 22776421

[ref-37] Pruessner JC Kirschbaum C Meinlschmid G : Two formulas for computation of the area under the curve represent measures of total hormone concentration versus time-dependent change. *Psychoneuroendocrinology.* 2003;28(7):916–931. 10.1016/s0306-4530(02)00108-7 12892658

[ref-38] Reiss F : Socioeconomic inequalities and mental health problems in children and adolescents: a systematic review. *Soc Sci Med.* 2013;90:24–31. 10.1016/j.socscimed.2013.04.026 23746605

[ref-39] Roberts AG Lopez-Duran NL : Developmental influences on stress response systems: implications for psychopathology vulnerability in adolescence. *Compr Psychiatry.* 2019;88:9–21. 10.1016/j.comppsych.2018.10.008 30466015

[ref-40] Rotenberg S McGrath JJ Roy-Gagnon MH : Stability of the diurnal cortisol profile in children and adolescents. *Psychoneuroendocrinology.* 2012;37(12):1981–1989. 10.1016/j.psyneuen.2012.04.014 22658393 PMC5760225

[ref-41] Smith AE Powers SI : Off-time pubertal timing predicts physiological reactivity to post-puberty interpersonal stress. *J Res Adolesc.* 2009;19(3):441–458. 10.1111/j.1532-7795.2009.00602.x 19823698 PMC2759532

[ref-70] Susman EJ Dockray S Granger DA : Cortisol and alpha amylase reactivity and timing of puberty: Vulnerabilities for antisocial behaviour in young adolescents. *Psychoneuroendocrinology.* 2010;35(4):557–569. 10.1016/j.psyneuen.2009.09.004 19819639 PMC2843813

[ref-42] Turner AI Smyth N Hall SJ : Psychological stress reactivity and future health and disease outcomes: a systematic review of prospective evidence. *Psychoneuroendocrinology.* 2020;114: 104599. 10.1016/j.psyneuen.2020.104599 32045797

[ref-43] Ullsperger JM Nikolas MA : A meta-analytic review of the association between pubertal timing and psychopathology in adolescence: are there sex differences in risk? *Psychol Bull.* 2017;143(9):903–938. 10.1037/bul0000106 28530427

[ref-44] Vliegenthart J Noppe G Van Rossum EF : Socioeconomic status in children is associated with hair cortisol levels as a biological measure of chronic stress. *Psychoneuroendocrinology.* 2016;65:9–14. 10.1016/j.psyneuen.2015.11.022 26708067

[ref-45] West BT : Analyzing longitudinal data with the Linear Mixed Models procedure in SPSS. *Eval Health Prof.* 2009;32(3):207–228. 10.1177/0163278709338554 19679634

[ref-46] Whelan E O’Shea J Hunt E : Evaluating measures of allostatic load in adolescents: a systematic review. *Psychoneuroendocrinology.* 2021;131: 105324. 10.1016/j.psyneuen.2021.105324 34198124

[ref-47] Worthman CM Dockray S Marceau K : Puberty and the evolution of developmental science. *J Res Adolesc.* 2019;29(1):9–31. 10.1111/jora.12411 30869841 PMC6961839

[ref-48] Zhang DD Fang J Zhang L : Pubertal recalibration of cortisol reactivity following early life parent-child separation. *J Affect Disord.* 2021;278:320–326. 10.1016/j.jad.2020.09.030 32979563

