# Supplementary material for: Assessing the stability of psychobiological stress reactivity during adolescence: mixed-effect modelling of cortisol responses to laboratory stressors
Source: HRB Open Res. 2025 Jan 24;7:26. Originally published 2024 Apr 25. [Version 2] doi: 10.12688/hrbopenres.13874.2 (PMC11776060; doi:10.12688/hrbopenres.13874.2)
Supplement: Peer review history

# Reviewer report: Reviewer response for version 2

**DOI:** 10.21956/hrbopenres.15328.r45055

**Published:** 2025-02-07

**Authors:** Jost Ulrich Blasberg

**Affiliations:** 1 Jena University Hospital, Friedrich-Schiller University, Jena, Germany

The authors present an interesting secondary study on cortisol reactivity across adolescence and its’ association with pubertal timing and tempo. This is a novel research question, as most studies have solely focused on the role of pubertal stage.

 Major concerns:

1. There are still numerous typos and grammatical errors in the manuscript. Please carefully proof read the manuscript.
2. The  bio-psychological and statistical methods are too short. Please provide a figure of the data-collection procedure to facilitate understanding. Please elaborate on how exactly you modelled your data.
3. The results section is very sparse. Please provide figures of your cortisol trajectories in the results section and if possible, a table including all model coefficients. No info on random variance is given. No model indices are given.

Minor concerns:

 Abstract

  

1. without more context the results in the abstract are confusing. What is meant by “Results of a linear mixed-effect model found there to be a significant difference in cortisol reactivity over time, indicating that cortisol stress reactivity did not remain stable during this time”? Time across the TSST? Time across adolescene?

 

1. “Additionally, results show children and adolescents who developed slower/quicker than peers displayed decreased stress reactivity” – maybe phrase it as pubertal tempo rather than slower/quicker?

Intro

  

1. “The interaction of parental relationships and pubertal timing has been observed in both boys and girls, with positive parenting…” – please elaborate on what is meant by “positive parenting”.
2. “It is important to note that while pubertal development and HPA maturation is associated with negative outcomes, some suggest that these should be treated as neither antecedents nor consequences of development, but rather as contributors to a system of accumulating risk, such as with allostatic load ( Joos *et al.*, 2018; Whelan *et al.*, 2021).” – I couldn’t quite grasp the meaning of this sentence, maybe rephrase? It is not clear why pubertal development and HPA maturation is associated with negative outcomes. Do you mean in the context of pubertal tempo?

Methods

1. “Of the 135 included in the final data set, thirty-three were removed due to missing data in cortisol reactivity or pubertal measured.” – do you mean pubertal status measured?
2. At the beginning of the methods section, please state clearly that data was collected in three ways. This would greatly facilitate understanding of the data collection procedure.
3. Please state the collection procedure of saliva (Salivettes?) and cortisol assessment (laboratory, immunoassay intra- and interassay variations)

Results

1. “We found that more rapid pubertal tempo was associated with a reduced cortisol reactivity profile, however the width of confidence interval limits meaningful inference.” If I understand this correctly, this is a main effect on the intercept of the model and not the time slope (i.e. reactivity across waves?). If this is true, this would mean that pubertal tempo had a significant effect on cortisol reactivity during the first wave, which sounds kind of paradoxical, as the tempo relates to the speed across all three waves, right? Unless the effect is on a grand mean of reactivity across all three waves. Please specify on how your coded your model intercept in the statistical methods and elaborate on this in the results!
2. The results section could be greatly improved by adding a figure of cortisol curves across the three timepoints.
3. Please report on some model statistics such as normality of residuals. Are your estimates (and especially confidence intervals) interpretable or skewed?
4. Were your independent variables z-standardized?
5. I would presume that pubertal timing and pubertal tempo are highly correlated. It might be a good idea to calculate separate models to assure no concerns of multicollinearity.

Discussion

1. “One strategy with potential to do this is to integrate different measures of pubertal timing ( Dorn *et al.*, 2006; Mendle, 2014), which would allow for.” – please finish this sentence.

Kudos:

1. I found the introduction to be concise while showing a clear understanding of the extent literature. The research question was also clearly developed and stated.
2. I found the calculation of pubertal timing via regression residuals quite fitting!

Is the work clearly and accurately presented and does it cite the current literature?

Yes

If applicable, is the statistical analysis and its interpretation appropriate?

Partly

Are all the source data underlying the results available to ensure full reproducibility?

Partly

Is the study design appropriate and is the work technically sound?

Partly

Are the conclusions drawn adequately supported by the results?

Yes

Are sufficient details of methods and analysis provided to allow replication by others?

No

Reviewer Expertise:

NA

I confirm that I have read this submission and believe that I have an appropriate level of expertise to confirm that it is of an acceptable scientific standard, however I have significant reservations, as outlined above.

# Reviewer report: Reviewer response for version 2

**DOI:** 10.21956/hrbopenres.15328.r45052

**Published:** 2025-01-28

**Authors:** Jessica Buthmann

**Affiliations:** 1 Stanford University, Stanford, California, USA

In this study the authors endeavored to test whether cortisol reactivity, as measured by the Trier Social Stress Test (TSST), was stable over a period of one year in a sample of adolescents. Although this is a worthwhile research question, this paper has three main issues. First, the results, which state that tempo was associated with cortisol change, are contradicted by the discussion, which states that neither timing nor tempo were associated with cortisol. Second, the TSST was not designed to be administered to the same participants multiple times, especially in such a short period of time. There are habituation issues to be considered. Third, the methods are lacking in detail that would provide greater context and make this study replicable.

 Abstract

- There is a typo in the last sentence of the Background section - the first world should probably be "The," not "This."
- "adolescent-aged children and adolescents" in the Methods section is odd, maybe remove "adolescent-aged."
- It is not clear from the abstract that the study uses data from more than one timepoint.
- This is not clear - is it slower or quicker? "children and adolescents who developed slower/quicker than peers displayed decreased stress reactivity"

 

 Introduction

- The paragraph that the following sentence concludes does not mention any social context, and is therefore a bit of a nonsequitor: "What these studies may indicate, is that the timing of puberty is a complex, highly socially contextual process that is closely linked with outcomes related to stress."
- This is not clear to me: "One recent systematic review of outcomes related to pubertal tempo found that while both boys and girls who experienced accelerated development had symptoms of depression during childhood and adolescence, there were distinct gender differences ( Cheng *et al.*, 2020). For instance, boys tended to experience more psychosocial difficulties due to accelerated development, for girls this was an opportunity to compensate for the impact of later onset puberty. "

 

 Methods

- "At the first wave of measurement, girls were aged 8, 10 or 12 years (Mean = 10.96, SD=1.66), and boys were aged 9, 11 or 13 years (Mean = 10.08, SD = 1.66)" this is repeated above and below Table 1. Can the authors clarify if/why 9 or 11 year old girls were not recruited, or 10 or 12 year old boys?
- The average amount of time between assessments should be clearly stated.
- It is difficult to interpret the pubertal timing and tempo variables. For context, can the authors provide information about the average pubertal stage at each wave by sex? It would be helpful for readers to know how much change occurred in the study period.
- The authors should explicity state how many times the Trier Social Stress Test was administered.
- How were outliers (especially in the cortisol data) handled?
- At which Wave(s) was the CBCL administered?
- Why was the Anxious/Depressed subscale used? Why not Internalizing or any of the other subscales?
- The statistics for covariates (SES, BMI, and sex) should be presented in Table 3.
- Did the authors check for multicollinearity between pubertal timing and pubertal tempo?
- Were there any significant differences in sex, SES, BMI, etc. between those who did and those who did not complete all waves of the assessment?

 

 Discussion

- This "There was no significant direct impact of either pubertal timing, or pubertal tempo on cortisol reactivity indicated in the results" contradicts the last sentence of the results: "We found that more rapid pubertal tempo was associated with a reduced cortisol reactivity profile." This is a huge issue.
- More discussion is needed about the meaningful difference between pubertal timing and tempo, and why one might be related to cortisol reactivity and not the other.
- A limitation to this study, which is not discussed, is that the TSST is not designed for repeated administration. In the Allen et al. 2017 paper cited by the authors, it is cautioned that HPA-axis reactivity in particular is subject to habituation with repeated administration. Given that the total time between Waves 1 and 3 is roughly a year, this seems especially problematic.

Is the work clearly and accurately presented and does it cite the current literature?

Partly

If applicable, is the statistical analysis and its interpretation appropriate?

Partly

Are all the source data underlying the results available to ensure full reproducibility?

Partly

Is the study design appropriate and is the work technically sound?

Partly

Are the conclusions drawn adequately supported by the results?

No

Are sufficient details of methods and analysis provided to allow replication by others?

No

Reviewer Expertise:

Child and adolescent development

I confirm that I have read this submission and believe that I have an appropriate level of expertise to state that I do not consider it to be of an acceptable scientific standard, for reasons outlined above.

# Reviewer report: Reviewer response for version 2

**DOI:** 10.21956/hrbopenres.15328.r44986

**Published:** 2025-01-27

**Authors:** Angelika Ecker

**Affiliations:** 1 Department of Child and Adolescent Psychiatry and Psychotherapy, University of Regensburg, Regensburg, Germany

The authors have put significant effort and dedication into revising the manuscript, which has greatly contributed to a clearer methodology and improved comprehensibility. With the implemented changes, I fully support the publication of the results.

Is the work clearly and accurately presented and does it cite the current literature?

Partly

If applicable, is the statistical analysis and its interpretation appropriate?

Yes

Are all the source data underlying the results available to ensure full reproducibility?

No

Is the study design appropriate and is the work technically sound?

Partly

Are the conclusions drawn adequately supported by the results?

Yes

Are sufficient details of methods and analysis provided to allow replication by others?

No

Reviewer Expertise:

I studied the multimodal stress reaction of healty adolescents of both sexes with different puberty stages to a TSST-C (original protocoll vs. a virtual adaptation).

I confirm that I have read this submission and believe that I have an appropriate level of expertise to confirm that it is of an acceptable scientific standard.

# Reviewer report: Reviewer response for version 2

**DOI:** 10.21956/hrbopenres.15328.r44985

**Published:** 2025-01-25

**Authors:** Megan R Gunnar

**Affiliations:** 1 Institute of Child Development, University of Minnesota, Minnesota, Minneapolis, USA

The authors were very responsive to my previous concerns. This is a nice paper.

Is the work clearly and accurately presented and does it cite the current literature?

Partly

If applicable, is the statistical analysis and its interpretation appropriate?

Yes

Are all the source data underlying the results available to ensure full reproducibility?

No

Is the study design appropriate and is the work technically sound?

Yes

Are the conclusions drawn adequately supported by the results?

Yes

Are sufficient details of methods and analysis provided to allow replication by others?

No

Reviewer Expertise:

I study the effects of early life stress on cortisol reactivity and regulation, often using the TSST.

I confirm that I have read this submission and believe that I have an appropriate level of expertise to confirm that it is of an acceptable scientific standard.

# Reviewer report: Reviewer response for version 1

**DOI:** 10.21956/hrbopenres.15210.r40365

**Published:** 2024-06-18

**Authors:** Angelika Ecker

**Affiliations:** 1 Department of Child and Adolescent Psychiatry and Psychotherapy, University of Regensburg, Regensburg, Germany

Thank you very much for the opportunity to review this manuscript on the stability of cortisol reactivity during pubertal development. The authors investigated the change in cortisol reactivity in children of both sexes over the course of a year using an acute stress response by the TSST-C.

 The manuscript is written in a clear and understandable style and with regard to cortisol reactivity in relation to the puberty timing, but also with regard to the underrepresented variable of pubertal tempo. Nevertheless, I have some crucial comments that make it difficult to publish this manuscript in its current form and should be addressed in a revision.

*General remarks*

- The youngest participants are 8 years old - for me, the term "adolescents" is somewhat inappropriate.
- Are the participants also off-time or off-tempo in their pubertal development? Could this have influenced the results?
- I wonder whether the increasingly early onset of puberty, without being premature puberty, could play a role in the sample analyzed here (e.g. doi: 10.1001/jamapediatrics.2019.5881).

*Introduction:*

- The theoretical background lacks something about the speed of change in cortisol reactivity that would justify the time period studied.

*Method*

- The final sample size is not clear from the text - according to the authors is N=102, but the participant chapter suggests N=111.
- Is there a theoretically based explanation for the choice of these age groups in the genders?
- The demographic sample description requires revision and, in particular, supplementation. I would suggest that age, sex, distribution in pubertal stages and SES, and BMI are presented for each wave. In addition, report the temporal variance between the waves. Perhaps also the anxiety levels with a reference range from the questionnaire in order to be able to assess how anxious the sample is.
- This could also clarify my subsequent question regarding why the boys' group is younger on average than the girls' group despite the higher age of inclusion
- You mention that chronic diseases have been ruled out - also other (non-chronic) diseases that could influence cortisol reactivity?
- In relation to pubertal development, only genital or breast development was used. Were the girls also controlled for menarche?
- You report that the CBCL "was administered by a researcher to the child", which would then be the self-report, i.e. the YSR, which, however, does not begin in the scope of application until the age of 11 and would therefore not be appropriate at this point. Alternatively, did the parents (CBCL) fill it out? Furthermore, it is not clear from the text at what wave anxiety was assessed and whether it was filled out repeatedly.
- In addition, I ask myself how confounded "anxiety" was with depression and how reliable and valid the subscale of a questionnaire on problem domains is. Furthermore, it is not clear from the theoretical background why this parameter was considered at all.

*Discussion*

- In my opinion, the end of the discussion could benefit from practical implications regarding the results, e.g. for stress research in adolescents during puberty, prevention programs during puberty (public health), or other areas.

Is the work clearly and accurately presented and does it cite the current literature?

Partly

If applicable, is the statistical analysis and its interpretation appropriate?

Yes

Are all the source data underlying the results available to ensure full reproducibility?

No

Is the study design appropriate and is the work technically sound?

Partly

Are the conclusions drawn adequately supported by the results?

Yes

Are sufficient details of methods and analysis provided to allow replication by others?

No

Reviewer Expertise:

I studied the multimodal stress reaction of healty adolescents of both sexes with different puberty stages to a TSST-C (original protocoll vs. a virtual adaptation).

I confirm that I have read this submission and believe that I have an appropriate level of expertise to confirm that it is of an acceptable scientific standard, however I have significant reservations, as outlined above.

# Response

**Published:** 2024-08-20

**Authors:** Jen O'Shea

**Affiliations:** School of Applied Psychology, University College Cork, Cork, County Cork, Ireland

Dear Dr Ecker, 

 I would like to thank you on behalf of the authors for your thoughtful and helpful comments. 

 Please our responses to these comments below. 

*General remarks*

- The youngest participants are 8 years old - for me, the term "adolescents" is somewhat inappropriate.

Thank you for your comment. The language used in the paper has been amended to state children and/or adolescents.

- Are the participants also off-time or off-tempo in their pubertal development? Could this have influenced the results?

Thank you for your comment.. The impact off-timing and off-tempo development is represented in Table 2. Oftentimes, studies will generate categories of early, on time, late, and slow, average, and fast tempo. This study had the opportunity to represent this in linear models where each participant is assessed relative to peers, and was informed by (Mendle et. al., 2019).  

- I wonder whether the increasingly early onset of puberty, without being premature puberty, could play a role in the sample analyzed here (e.g. doi:10.1001/jamapediatrics.2019.5881).

Thank you for this comment. The global trend of decreasing age of puberty likely will impact the calculation of pubertal timing (and possibly tempo) in any research sample, and are an intriguing and challenging aspect of understanding cohort and generation effects, including on pubertally-mediated changes in  cortisol responses to stress. However, as the pubertal development were calculated within sample, relative differences cannot be considered with this data alone.

*Introduction:*

- The theoretical background lacks something about the speed of change in cortisol reactivity that would justify the time period studied.

Due to the very rapid development of the Hypothalamus and related endocrine systems, and previous research on the recalibration of stress responses, adolescence is of particular interest for studying change in cortisol reactivity. This perspective has now been added in the introduction.

*Method*

- The final sample size is not clear from the text - according to the authors is N=102, but the participant chapter suggests N=111.

Thank you for noting the need for clarity, we have amended the Method section  to describe the total data set size, and final number included for analysis.

  

- Is there a theoretically based explanation for the choice of these age groups in the genders?

Thank you for your comment.. This difference in age is in effort to capture children/adolescents at similar stages of development, given that girls begin puberty at an earlier age than boys. This has now been added to the methods section.

- The demographic sample description requires revision and, in particular, supplementation. I would suggest that age, sex, distribution in pubertal stages and SES, and BMI are presented for each wave. In addition, report the temporal variance between the waves. Perhaps also the anxiety levels with a reference range from the questionnaire in order to be able to assess how anxious the sample is.

Thank you for your comment. Additional details have been inserted into the manuscript to describe these factors, and to previous papers that have elaborated on the sample.

- This could also clarify my subsequent question regarding why the boys' group is younger on average than the girls' group despite the higher age of inclusion

The mean age of boys in the sample is due to a higher number of younger boys being recruited. Some additional details have been added to describe the ages of participants across waves.

- You mention that chronic diseases have been ruled out - also other (non-chronic) diseases that could influence cortisol reactivity?

Thank you for your comment. Yes, in addition to chronic diseases, and non-chronic disease that affect the endocrine system, participants were also excluded based on factors such as medication, and this has not been described in the Method.

- In relation to pubertal development, only genital or breast development was used. Were the girls also controlled for menarche?

Thank you for your comment. These metrics were selected guided by the research by Mendle & colleagues (2019). While menarche is often used as a metric for pubertal development calculation, We did not include it as a control as it may have distorted the timing of general pubertal development in the calculation.

- You report that the CBCL "was administered by a researcher to the child", which would then be the self-report, i.e. the YSR, which, however, does not begin in the scope of application until the age of 11 and would therefore not be appropriate at this point. Alternatively, did the parents (CBCL) fill it out? Furthermore, it is not clear from the text at what wave anxiety was assessed and whether it was filled out repeatedly.

Thank you for your comment. The CBCL was administered to young people where appropriate, however, all parents / guardians completed the parent version. The parent measure was used for calculation as it was available for all participants. This was assessed at each wave of development, and mean scores at each wave can be seen in Table 1

- In addition, I ask myself how confounded "anxiety" was with depression and how reliable and valid the subscale of a questionnaire on problem domains is. Furthermore, it is not clear from the theoretical background why this parameter was considered at all.

Thank you for your comment. There may have been some confounding between anxiety and depression. However, as the remit of this study as the impact of pubertal development on cortisol reactivity stability, these measures were used to account for variance in our model across each time point. These values were selected as anxiety, depression, and other factors such as this can impact on cortisol responses directly, in wither heightening or blunting the response.

*Discussion*

- In my opinion the end of the discussion could benefit from practical implications regarding the results e.g. for stress research in adolescents during puberty prevention programs during puberty (public health) or other areas.

Thank you for your comment and helpful suggestion, and we have now included this in the Discussion.

 Once again I would like to extend our gratitude for your comments and suggestions. 

 Warm regards,

 Jen O'Shea

# Reviewer report: Reviewer response for version 1

**DOI:** 10.21956/hrbopenres.15210.r40037

**Published:** 2024-06-04

**Authors:** Megan R Gunnar

**Affiliations:** 1 Institute of Child Development, University of Minnesota, Minnesota, Minneapolis, USA

Overall, this is an interesting re-analysis of data on cortisol reactivity previously presented by Ji et al,   In this re-analysis, the purpose is to :

*This purpose of this study was to examine whether patterns of cortisol reactivity remained stable across one year of pubertal development, and whether variations in pubertal*

*development impacted on this stability”*

 Notably the stability of the cortisol response was thoroughly analyzed in the Ji et al paper. What is novel about the present analysis is the assessment of timing and tempo of puberty in relation to cortisol reactivity and change. This needs to be made very clear. It took me some mining of the article to figure out the link with Ji et al. Although the analytic strategies were different between these two studies, it is inappropriate to present the question of stability without acknowledging that the present study just examines stability different and arrives at largely the same conclusion. Again, the timing and tempo questions are new and fine to focus on.

 There are some other issues I have with this paper. They can be fixed.

 Please clarify the N of the study. First it says there were 136 participants. Then 89+26=111 participants, then 102. How many participants were there? I presume 102 as this is what they ended up with in Ji et al.

 Please provide a table that includes the race and ethnicity of the participants, ages (mean, range) at each wave, the time between assessments (mean and range). From Ji et al it is clear that nearly all (89%) are white, non-hispanic.

 Please clarify the time period over which these data were collected. In one place it says 3 years. In another it says that there were 9 mos between TSST-C assessment. If the latter is true then Wave 1=0, Wave 9=0+9, Wave=0+9+9 (or 18 months from the beginning assessment to the end). In the Ji et al paper (which I think is the same data set) it states that there were 6 months between assessments.

Is the work clearly and accurately presented and does it cite the current literature?

Partly

If applicable, is the statistical analysis and its interpretation appropriate?

Yes

Are all the source data underlying the results available to ensure full reproducibility?

No

Is the study design appropriate and is the work technically sound?

Yes

Are the conclusions drawn adequately supported by the results?

Yes

Are sufficient details of methods and analysis provided to allow replication by others?

No

Reviewer Expertise:

I study the effects of early life stress on cortisol reactivity and regulation, often using the TSST.

I confirm that I have read this submission and believe that I have an appropriate level of expertise to state that I do not consider it to be of an acceptable scientific standard, for reasons outlined above.

# Response

**Published:** 2024-08-20

**Authors:** Jen O'Shea

**Affiliations:** School of Applied Psychology, University College Cork, Cork, County Cork, Ireland

Dear Professor Gunnar, 

 I would like to thank you for your time, and for your very helpful comments. 

 Please find individual responses to each of your comments/suggestions below. 

- Notably the stability of the cortisol response was thoroughly analyzed in the Ji et al paper. What is novel about the present analysis is the assessment of timing and tempo of puberty in relation to cortisol reactivity and change. This needs to be made very clear. It took me some mining of the article to figure out the link with Ji et al.

Thank you for your observation and helpful comment. We have revised the introduction to explicitly state that the remit of the study is in the potential role of off-time and off tempo pubertal development on cortisol reactivity.

- Although the analytic strategies were different between these two studies, it is inappropriate to present the question of stability without acknowledging that the present study just examines stability different and arrives at largely the same conclusion.

The authors agree; the analytic methods of previous studies was not explicitly described and acknowledged  in the original paper. This has been amended in the methods section of the paper. It is likely that our study arrived at the same conclusion due to pattern of increasing and/or varying responses and recovery to stressors. Additionally, the previous study in question examined cortisol response and recovery, as opposed to the present study that examined response only. It is because of this that AUCi was chosen as the preferred method of calculation.

- Please clarify the N of the study. First it says there were 136 participants. Then 89+26=111 participants, then 102. How many participants were there? I presume 102 as this is what they ended up with in Ji et al.

Thank you for noting the error, it has been corrected in the revised paper to state that of 135 participants, 33 were removed due to missing data.

- Please provide a table that includes the race and ethnicity of the participants, ages (mean, range) at each wave, the time between assessments (mean and range). From Ji et al it is clear that nearly all (89%) are white, non-hispanic.

Thank you for your comment. This table has now been added to the manuscript, as well as direction to find further details of the sample.

- Please clarify the time period over which these data were collected. In one place it says 3 years. In another it says that there were 9 mos between TSST-C assessment. If the latter is true then Wave 1=0, Wave 9=0+9, Wave=0+9+9 (or 18 months from the beginning assessment to the end). In the Ji et al paper (which I think is the same data set) it states that there were 6 months between assessments.

Thank you for noting that this is unclear, we have  amended the manuscript to state more clearly that data collection waves took place over 1 year, at 6 month intervals (baseline, +6 months, +12 months). A table has also been added noting the number of days elapsed between waves.

 Once again on behalf of the the authors, I would like to thank you for your comments. 

 Warm regards,

 Jen O'Shea
